# BADGE, a synthetic antagonist for PPARγ, prevents steroid-related osteonecrosis in a rabbit model

**DOI:** 10.1186/s12891-018-2050-6

**Published:** 2018-04-27

**Authors:** Na Yuan, Jia Li, Meng Li, Wenchen Ji, Zhaogang Ge, Lihong Fan, Kunzheng Wang

**Affiliations:** 1 Department of Ultrasonography, The First Affiliated Hospital of Xi’an Jiaotong University, Xi’an, Shaanxi 710061 People’s Republic of China; 2 Department of Orthopedics, The First Affiliated Hospital of Xi’an Jiaotong University, 277 West Yanta Road, Xi’an, Shaanxi 710061 People’s Republic of China; 3 Department of Joint Surgery, Honghui Hospital of Xi’an Jiaotong University, Xi’an, Shaanxi 710054 People’s Republic of China; 4 The first department of Orthopedics, The Second Affiliated Hospital of Xi’an Jiaotong University, Xi’an, Shaanxi 710004 People’s Republic of China

**Keywords:** Osteonecrosis, Adipogenesis, Ppar γ, Osteogenesis

## Abstract

**Background:**

It was indicated that inhibition of PPARγ probably represents a novel therapy for steroid-related osteonecrosis. In this study, we investigated the preventive effects of PPARγ inhibition on steroid-related osteonecrosis in a rabbit model.

**Methods:**

Rabbits were randomly divided into three groups (normal group, model group and BADGE group). Osteonecrosis was induced in rabbits in the model group and the BADGE group. The BADGE group also received bisphenol a diglycidyl ether(BADGE), a PPARγ antagonist, for 6 weeks.

**Results:**

Histopathological results indicated that rabbits treated with BADGE exhibited significantly reduced osteonecrotic changes, incidence of osteonecrosis and bone marrow adiposity. Furthermore, BADGE-treated rabbits exhibited reduced intraosseous pressure and increased femoral blood perfusion. Micro-computed tomography and bone histomorphometry indicated that the BADGE group exhibited significantly improved bone quality and mineral appositional rate compared with the model group. Furthermore, the BADGE group showed a significant increase in circulating levels of the bone formation marker osteocalcin and reduced levels of the bone resorption marker TRACP. Overall, BADGE-treated rabbits exhibited reduced marrow adiposity concomitant with improved bone formation.

**Conclusions:**

In conclusion, these observations demonstrated that pharmacological inhibition of PPARγ might represent an effective therapy for steroid-related osteonecrosis in the near future.

## Background

Hypertrophy of bone marrow fat cells, which causes increased intraosseous pressure and decreased blood flow, plays crucial roles in the development of steroid-related osteonecrosis [1–3]. Glucocorticoids promote adipogenic differentiation of marrow stromal cells and inhibit their osteogenic differentiation in vitro [4]. The promoted adipogenesis also acts as a predominantly negative regulator of the bone marrow microenvironment [5] and promotes osteoclast differentiation [6, 7]. These features are potentially related to steroid-related osteonecrosis. Core decompression, which reduces the intra-medullary pressure, was formerly the most commonly used method to treat steroid-related osteonecrosis of the femoral head. However, the efficacy of the technique is controversial, and it has little effects on inhibiting bone marrow adipogenesis and fat cell hypertrophy [8].

Increasing evidence demonstrates that peroxisome proliferator-activated receptor γ(PPARγ), which is a specific transcription factor that plays important roles in adipogenic differentiation, is an important factor in the development of steroid-related osteonecrosis [9–11]. In vivo and in vitro studies indicate that steroids increase the expression of the PPARγ gene, which leads to induced adipogenic differentiation and repressed osteogenic differentiation [10, 12]. Correspondingly, by suppressing PPARγ, several methods are employed to prevent steroid-related osteonecrosis [9, 10].

Bisphenol a diglycidyl ether(BADGE), a synthetic antagonist for PPARγ, inhibits bone marrow adipogenesis both in vitro and in vivo [13, 14]. In addition, BADGE promotes osteoblastogenesis and bone formation, and reduces marrow adiposity [15]. Based on the abovementioned studies, the utilization of BADGE might offer a promise method for preventing steroid-related osteonecrosis by reducing adipogenesis and intraosseous pressure, improving local blood perfusion of the femoral head, inhibting osteoclastogenesis and promoting osteogenesis.

In the present study, we investigated the preventive effects of BADGE on steroid-related osteonecrosis in a rabbit model and explored the mechanism involved.

## Methods

### Animals and treatments

After approval from the Animal Ethical Committee of Xi’an Jiaotong University, 36 28-week-old male New Zealand white rabbits (4 ± 0.5 kg) from the Experimental Animal Center of Xi’an Jiaotong University, China were included in this study. All experimental animals were housed under standard conditions.

Rabbits were randomized into 3 groups: the normal group (*N* = 12), the model group (N = 12) and the BADGE group (N = 12). Osteonecrosis of femurs was induced using methods presented in the previously published protocols [16]. Briefly, one injection of 10 μg/kg body weight of lipopolysaccharide (Sigma, St. Louis, MO, USA) was administered intravenously. Twenty-four hours later, three injections of 20 mg/kg body weight of methylprednisolone (Pfizer, USA) were administered intramuscularly, at a time interval of 24 h. Osteonecrosis gradually developed in femurs 6 weeks after injection of methylprednisolone. In the model group, the rabbits were treated with vehicle. In the BADGE group, the rabbits received treatment for a total of 6 weeks with BADGE(daily intra-peritoneal injection of 10 mg/kg in 15% DMSO). The doses and durations of treatment of BADGE were determined based on a previously published study [15]. Body weight of the rabbits were measured each week.

### Histopathology

The rabbits were sacrificed with an overdose of pentobarbital sodium and bilateral thighbones were dissected for hematoxylin and eosin(HE) staining 6 weeks after the last injection of methylprednisolone. Briefly, bone samples were fixed with 10% neutral-buffered formalin and decalcified with 10% ethylene diamine tetraacetic acid for 6 weeks. After the samples were embedded in paraffin, the specimens were cut along the coronal plane into 4-μm-thick sections and stained with HE. The evaluation criteria of osteonecrosis were based on a published study by Yamamoto et al. [17]. During the observation of slides, the following parameters including incidence of osteonecrosis, rate of empty lacunae, average diameter of fat cells and average fat cell area were assessed by two independent authors who were blind according to previous studies [16, 18–20].

### Biochemical analysis

Blood samples were obtained at week 6 of treatment, and blood serum was prepared from each sample by centrifugation for 5 min at 3000 rpm. Serum was stored frozen at − 20 °C. To assess osteoblastic activity, osteocalcin (OCN) which is a serum marker of bone formation, was measured using an immunoradiometric kit (Beifang BioengineeringInstitute, Beijing, China) according to the manufacturers’ instructions. Serum TRACP, marker of bone resorption, was quantified using a TRACP assay kit(Nanjing JianchengBioengineering Institute, Nanjing, China).

### Western blot analysis

The bone samples were crushed under liquid nitrogen conditions using mechanical disruption technique. The protein extracts were obtained by incubation in RIPA buffer at 4 °C for 30 min. Solutions were then centrifuged at 12000 rpm for 20 min at 4 °C to remove insoluble material. Protein concentrations were determined using the BCA assay and the samples were stored at − 80 °C. For Western blot analysis, the samples were dissolved in SDS electrophoresis buffer (Bio-Rad, Hercules, CA, USA) and loaded on SDS-PAGE gels and transferred onto a PVDF membrane filters. The filter was blocked for 1 h and then incubated overnight at 4 °C with primary antibodies against osteocalcin, Runx2, adipocyte fatty acid binding protein2(aP2) and β-actin(Santa-Cruz, USA), followed by incubation with the corresponding secondary antibodies. The protein bands were quantitatively analyzed by use of an image analysis system (NIH Image, Version 1.61).

### PCR analysis

After the bone samples were crushed under liquid nitrogen conditions using a mechanical disruption technique, total RNA was extracted using the TRIzol reagent (Invitrogen, CA, USA) according to the manufacturer’s specifications. cDNA was synthesized from 2 μg of total RNA using the Revert Aid™ First Strand cDNA Synthesis Kit (Fermentas, Canada) as described by the manufacturer. The relative levels of targeted gene mRNA transcripts were determined by real-time quantitative RT-PCR using SYBR Green 1 PCR master mix (TaKaRa, Japan). The ∆∆Ct method was used to quantify the relative expression of each gene. PCR primers with the following sequences were commercially designed by Shengong Co. Ltd., Shanghai, China: GAPDH, forward: 5’-CCACTTTGTGAAGCTCATTTCCT-3′, reverse: 5’-TCGTCCTCCTCTGGTGCTCT-3′; osteocalcin, forward: 5’-TCACTCTTGTCGCCCTGCT-3′, reverse: 5’-CCTCCCTCTTGGACACGAA-3′; Runx2, forward: TGACCGCAGACATAATCCAT, reverse: GCCACTTTCGGAACAGAGAT; CCAAT/enhancer-binding protein β(CEBPβ), forward: CTACTACGAGGCGGACTGCT, reverse: GTACGGGCTGAAGTCGATG; aP2, forward: CGATAAACTGGTGGTGGAATGC, reverse: CCCGGGCTTATGCTCTTTCA. Real-time PCR was performed for 40 cycles of 95 °C for 15 s, an annealing temperature (60 °C for GAPDH, CEBPα and aP2, and 65 °C for osteocalcin and Runx2) for 30 s, and 72 °C for 30 s.

### Micro-computed tomography (μCT) analysis

μCT (eXploreLocus SP, GE, USA) was performed as described previously [20] to non-invasively evaluate bone structure using MS-8 system, which is a high-resolution system with a resolution of 14 μm per voxel, 6 weeks after the last injection of methylprednisolone. Briefly, a cylindricalregion of interest (ROI) in the trabecular region of the femoral head bone was selected. Images were reconstructed into 3-D volumes. The bone mineral density (BMD), bone volume fraction (BVF), trabecular separation (Tb.Sp), trabecular thickness (Tb.Th), and trabecular number (Tb.N) were calculated then.

### Dynamic histomorphometry analysis

Tetracycline and calcein labelling was performed to analyse dynamic histomorphometry. Briefly, rabbits were injected intramuscularly with 50 mg/kg of tetracycline 10 days before sacrifice and 5 mg/kg of calcein (Sigma) 3 days before sacrifice. Then, the thighbones were dissected out, fixed in 70% ethanol, dehydrated, and embedded undecalcified in methylmethacrylate. Then, 10-μm sections were cut, and the unstained sections were viewed using a fluorescence microscope. The mineral appositional rate (MAR, μm/day) was measured. MAR was defined as the mean distance between two fluorescent labels divided by the number of days between labels.

### Measurements of intraosseous pressure

Intraosseous pressure in the proximal femur of all rabbits was determined at week 6. The animals were anesthetized with 30 mg/kg of pentobarbital sodium. A standard lateral approach to expose the proximal aspect of the right femur was made under sterile conditions as described in previous reports [1, 21]. To create a drill hole, a drill with an outer diameter of 1.0 mm was inserted from the outer cortex 2.5 cm distal to the proximal end of the greater trochanter. The hole was then connected to a pressure transducer via a polyethylene catheter filled with heparinized saline. The pressure transducer was connected to a blood pressure amplifier (FY-2; ChengDu; China) for intraosseous pressure measurements. When the pressure turned steady, the steady recording was obtained as the intraosseous pressure. The systemic arterial pressure was measured simultaneously via an arterial catheter in the rabbit’s ear.

### Blood perfusion

To analyse blood perfusion, dynamic contrast-enhanced MRIs for the bilateral proximal femora were performed 6 weeks after the injection of MPS using a 3.0 T superconducting system (ACS-NT Intera, Philips Healthcare, The Netherlands) described previously [16, 22]. As the contrast agent, 0.8 mmol per kg of body weight of gadopentetatedimeglumine (Gd-DTPA, Magnevist, Schering, Berlin, Germany) was rapidly injected manually via a previously placed 21-gauge intravenous catheter in the right ear vein, followed by a 5-mL saline flush. A set of DCE-MRI was obtained soon after Gd-DTPA injection, and the signal intensity (SI) was measured. SI for each femur was plotted against time to create a time-signal intensity curve, and the perfusion index ‘maximum enhancement’ was calculated. Maximum enhancement was defined as (SI_max_ − SI_base_)/SI_base_ × 100%. SI_base_ was the baseline signal intensity obtained before Gd-DTPA injection, and SI_max_ was the maximum signal intensity after Gd-DTPA injection.

### Statistical analysis

All data were presented as the mean ± standard deviation (SD). One-way analysis of variance was performed to analyse the statistical data, and the Chi-square test was performed for the count data. The SPSS 17.0 software was used for analysis. In all experiments, a value of *p* < 0.05 was considered significant.

## Results

1. Treatment of rabbits with PPARγ inhibitors reduced osteonecrotic changes.

**Fig. 1:**
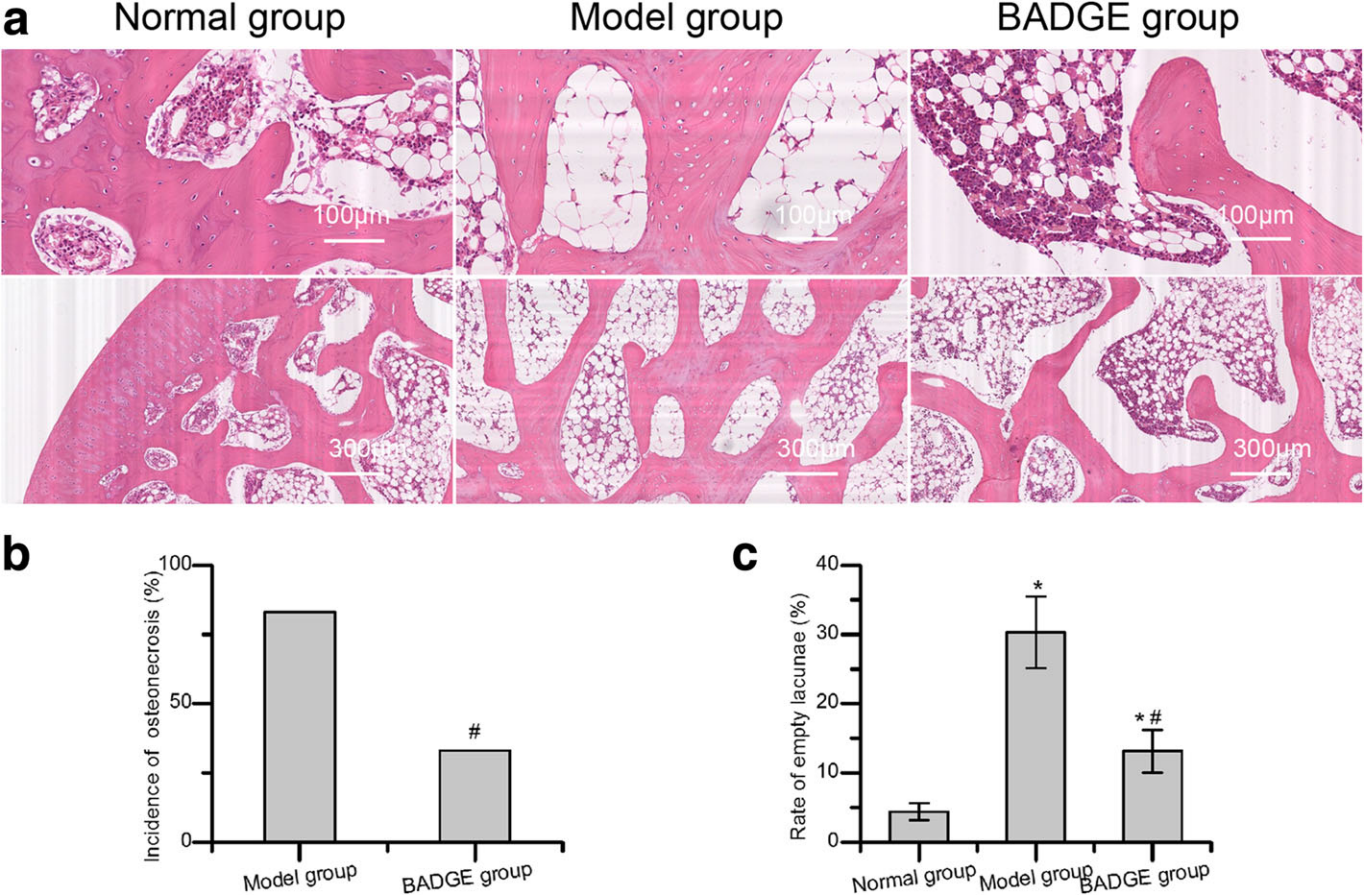
Treatment with PPARγ inhibitors reduced osteonecrotic changes and osteonecrosis incidence. **a** Histopathological observations. Normal bone tissue and typical osteonecrosis were observed in the normal group and the model group, respectively. In the model group, diffuse osteocytes in the trabeculae exhibited empty lacunae or pyknotic nuclei. In the BADGE group, slight osteonecrosis and fewer empty lacunae were observed. **b** A reduced osteonecrosis incidence was observed in the BADGE group compared with the model group (*P* < 0.05). **c** The rate of empty lacunae in the BADGE group was significantly reduced compared with the model group, and the significance was statistically significant (*P* < 0.05,*n* = 5). *P < 0.05 versus normal group; #*P* < 0.05 versus model group

No rabbits died in all groups. The body weight change did not differ between the model group and the BADGE group throughout the experimental period. Obvious osteonecrosis was observed in the model group (Fig. 1) with the diffuse presence of empty lacunae or pyknotic nuclei of osteocytes within the bone trabeculae accompanied by surrounding bone marrow cell necrosis or fat cell necrosis. In the BADGE group, slight osteonecrosis was observed. The incidence of osteonecrosis in the BADGE group was 33% (4/12) which was lower than 83% (10/12) in the model group (*P* < 0.05). In addition, the rate of empty lacunae in the BADGE group was significantly reduced compared with the model group (*P* < 0.05).

2. BADGE administration decreased bone marrow adiposity and intraosseous pressure, and promoted femoral blood perfusion.

**Fig. 2:**
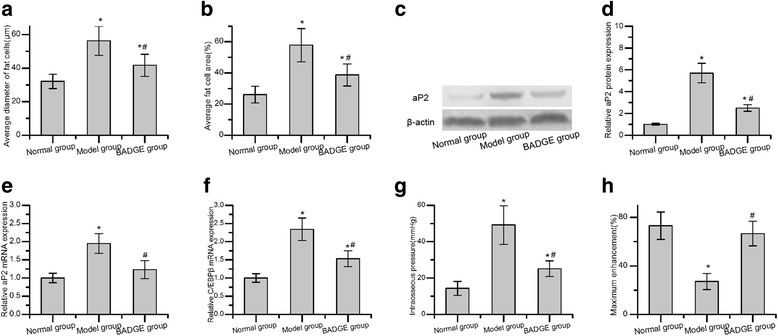
Treatment with PPARγ inhibitors reduced marrow adiposity and intraosseous pressure, and promoted femoral blood perfusion. **a** Compared with the model group, the average diameter of fat cells in the BADGE group decreased (*n* = 5). **b** The average fat cell area in the BADGE group was significantly reduced compared with the model group (*n* = 5). (**c** and **d**) BADGE treatment significantly decreased protein expression of the adipogenic transcription factor aP2 (*n* = 3). (**e** and **f**) Treatment with BADGE significantly decreased mRNA levels of the adipogenic transcription factors aP2 and CEBP (*n* = 3). **g** A lower intraosseous pressure was observed in the BADGE group compared with the model group (*n* = 5). **h** Blood perfusion, as indicated by dynamic contrast-enhanced MRIs, was significantly higher than that in the model group (*n* = 5). *P < 0.05 versus normal group; #*P* < 0.05 versus model group

As shown in Fig. 1a, marrow fat was significantly reduced in rabbits treated with BADGE compared with those in the model group. Both the average diameter and average area of fat cells were significantly reduced in the BADGE group compared with the model group (Fig. 2a and b). Furthermore, we detected the local expression of adipogenic factors, including CEBP and aP2. Both the protein and mRNA level of aP2 and the mRNA level of CEBP in the BADGE group were significantly reduced compared with the model group (Fig. 2c-f). Bone marrow adiposity plays essential roles in increasing intraosseous pressure and reducing blood flow [1–3]. In accordance with the decreased bone marrow adiposity, the intraosseous pressure in the proximal femur of rabbits in the BADGE group declined compared with the model group (Fig. 2g). In addition, blood perfusion to the proximal femora, which was measured by dynamic-contrast enhanced MRIs, was significantly enhanced in the BADGE group compared with the model group (Fig. 2h).

3. BADGE administration increased bone mass and stimulated bone formation

**Fig. 3:**
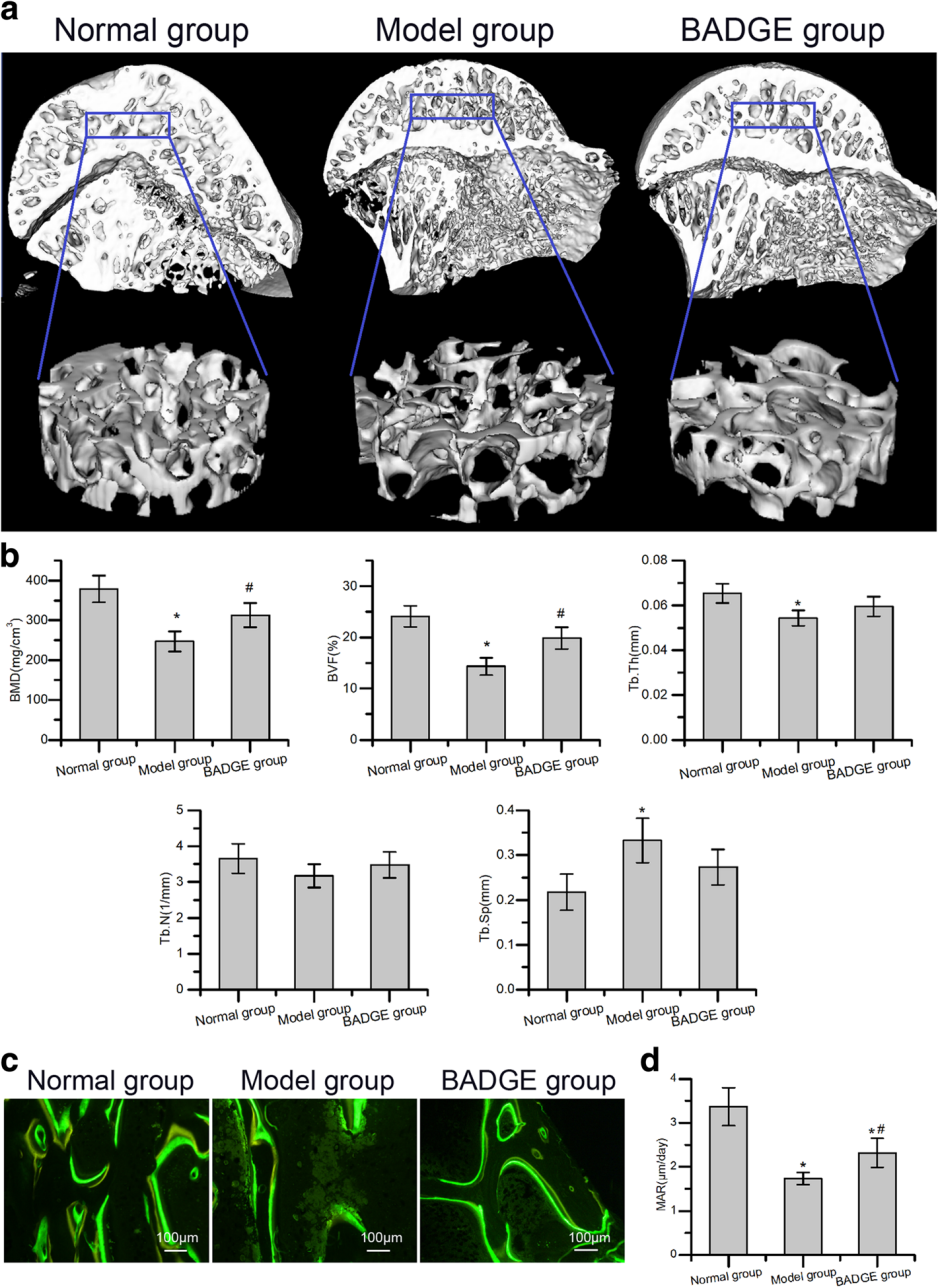
Treatment with PPARγ inhibitors ameliorated microstructural parameters and mineral apposition rate of trabecular bone. **a** Representative 3-D structure of femoral head of each group 6 weeks after the induction of osteonecrosis. **b** Quantitative analysis revealed significant reductions in BMD, BVF and Tb.Th and a significant increase in Tb.sp. in the model group compared with the normal group. Compared with the model group, the BADGE group exhibited significantly increased BMD and BVF (*n* = 5). **c** Representative tetracycline and calcein labelling images. **d** Quantitative analysis revealed a significantly increased mineral apposition rate in the BADGE group compared with the model group (*n* = 5). *P < 0.05 versus normal group; #*P* < 0.05 versus model group

**Fig. 4:**
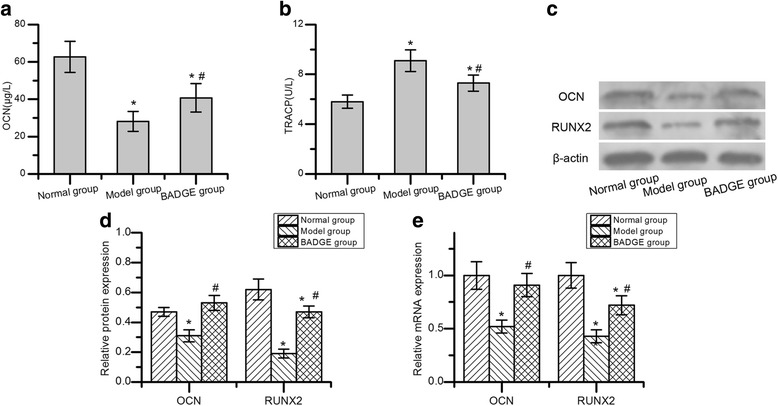
Treatment with PPARγ inhibitors increased the serum osteocalcin (OCN) concentration, decreased the serum TRAP concentration and promoted expression of the osteogenic transcription factors. **a** The concentration of osteocalcin in the BADGE group was significantly increased compared with the model group. **b** In contrast to increased serum osteocalcin, the TRAP concentration was reduced in the BADGE group compared with the model group (*n* = 5). **c**-**e** BADGE treatment significantly increased protein and mRNA expression of the osteogenic transcription factors osteocalcin and Runx2 (*n* = 3). *P < 0.05 versus normal group; #*P* < 0.05 versus model group

Micro-CT and undecalcified histology (Fig. 3) revealed evidence of increased trabecular bone formation in rabbits treated with the PPARγ inhibitor BADGE compared with those in the model group. Micro-CT analysis indicated a significant increase in bone relative to bone mineral density and bone volume fraction in the region of interest (Fig. 3b). Dynamic histomorphometry analysis for tetracycline and calcein labelling revealed increased mineral apposition rates (MAR) in trabecular bone in the rabbits receiving PPARγ inhibitors compared with those in the model group (Fig. 3d). The anabolic effect of PPARγ inhibitors on bone was further examined by quantifying the circulating concentrations of markers of bone formation (osteocalcin) and bone resorption (TRAP). A significantly increased concentration of osteocalcin was observed in the BADGE group compared with the model group (Fig. 4a). In contrast to increased serum osteocalcin, serum TRAP was reduced in the BADGE group compared with the model group (Fig. 4b). These data suggested coupling of bone formation and resorption. In contrast to the decreased expression of CEBP and aP2, the expression of osteocalcin and Runx2 in the BADGE group was significantly increased compared with the model group (Fig. 4c-f).

## Discussion

In this study, we explored the protective effects of BADGE on steroid-related osteonecrosis in rabbits. Our results indicated that PPARγ inhibition prevented steroid-related osteonecrosis, and the effect was in part owing to reduced marrow fat infiltration. The reduced bone adiposity was accompanied by an increase in bone mass and femoral blood perfusion and associated with decreased transcription of adipogenic genes and increased transcription of osteogenic genes.

Hypertrophy of fat cells and adipogenic differentiation of BMSCs induced by steroids is potentially associated with steroid-related osteonecrosis [1–3]. Previous studies have demonstrated that PPARγ is closely related to the induction of adipogenic differentiation and plays important roles in the development of steroid-related osteonecrosis [10, 12]. Steroids can also stimulate the expression of PPARγ mRNA both in vivo and in vitro, and increased PPARγ expression can reduce osteogenic differentiation, induce adipogenic differentiation, promote bone marrow adiposity, rise intraosseous pressure, block local blood perfusion, and finally lead to osteonecrosis. Correspondingly, downregulation of PPARγ expression might induce osteogenic differentiation, reduce adipogenic differentiation, inhibit bone marrow adiposity, decrease intraosseous pressure, promote local blood perfusion and eventually prevent osteonecrosis.

As a synthetic antagonist for PPARγ, bisphenol a diglycidyl ether (BADGE) inhibits adipogenic differentiation in vitro and prevents bone marrow adiposity in vivo [13, 14].

Botolin S and McCabe LR found that BADGE treatment prevents diabetes-induced hyperlipidemia, effectively blocks diabetes type I-induced bone adiposity and reduces the induction of adipogenic gene expression in insulin-deficient diabetic BALB/c mice [14]. Similar results were presented in our study. In the present study, we found that in rabbits treated with both BADGE and steroids, bone marrow adiposity was significantly reduced compared with that in rabbits treated only with steroids. In addition, the expression of adipogenic factors, including CEBP and aP2, was significantly repressed. Furthermore, we detected intraosseous pressure and femoral blood perfusion given that bone marrow adiposity is closely related to intraosseous pressure and blood flow. Similar to the reduced bone marrow adiposity, BADGE administration reduced the intraosseous pressure in the proximal femur and increased blood perfusion to the proximal femora.

A reciprocal relationship exists between osteogenic and adipogenic differentiation of mesenchymal stem cells. The imbalance between the osteogenic and adipogenic differentiation of MSCs [12] is involved in the occurrence of steroid-related osteonecrosis. PPARγ plays a vital role in modulating differentiation of mesenchymal stem cells, stimulating adipocyte development at the expense of osteoblast differentiation [23]. Hence, PPARγ may serve as a valuable target for methods intended to enhance bone mass. Akune T et al. demonstrated that heterozygous PPARγ-deficient mice exhibit high bone mass and reduced marrow adiposity as well as increased osteoblast number and bone formation rates in mice [24]. In accordance with the findings of Akune T et al., Gustavo Duque and his colleagues indicated that pharmacological inhibition of PPARγ via BADGE increases bone mass and osteoblastogenesis and stimulates bone formation in male C57BL/6 mice [15]. However, Botolin S and McCabe LR found that pharmacological inhibition of PPARγ by BADGE can not promote expression of osteoblast markers and bone density in diabetic mice [14]. In our study, we explored the effect of BADGE on bone mass, bone formation rates, and expression of osteogenic transcription factors in steroid-related osteonecrosis animal models. Rabbits treated with both BADGE and steroid exhibited increased bone mass and mineral apposition rates. These effects were associated with elevated expression of osteogenic transcription factors, including osteocalcin and Runx2, in the BADGE-treated rabbits.

One limitation of the present study was that we limited the therapeutic dose of BADGE to 10 mg/kg. We were unable to determine the optimal level of BADGE. Thus, in the near future, we will conduct additional studies to determine the optimal level of BADGE that can prevent the development of osteonecrosis.

## Conclusions

In summary, our data indicated that PPARγ inhibition by BADGE significantly decreases the incidence of osteonecrosis in steroid-treated rabbits. The present study also provided evidence to suggest that BADGE might prevent steroid-related osteonecrosis by reducing bone marrow adiposity and intraosseous pressure, promoting local blood perfusion, decreasing the expression of adipogenic transcription factors and increasing the expression of osteogenic transcription factors.

## Abbreviations

aP2: Adipocyte fatty acid binding protein 2
BADGE: Bisphenol a diglycidyl ether
BMD: Bone mineral density
BVF: Bone volume fraction
CEBPβ: CCAAT/enhancer-binding protein β
LPS: Lipopolysaccharide
MAR: Mineral appositional rate
MPS: Methylprednisolone
OCN: Steocalcin
PPARγ: Peroxisomal proliferator-activated receptor γ
ROI: Region of interest
SI: Signal intensity
Tb.N: Trabecular number
Tb.Sp: Trabecular separation
Tb.Th: Trabecular thickness
μCT: Micro-computed tomography
